# Microbiologic Spectrum and Antibiotic Susceptibility Pattern among Patients with Urinary and Respiratory Tract Infection

**DOI:** 10.1155/2014/682304

**Published:** 2014-06-29

**Authors:** Maryam Sotoudeh Anvari, Mohammad Naderan, Mohammad Ali Boroumand, Saeed Shoar, Robab Bakhshi, Morteza Naderan

**Affiliations:** ^1^Department of Surgical and Clinical Pathology, Tehran Heart Center, Tehran University of Medical Sciences, Tehran 1411713138, Iran; ^2^Department of Research, Tehran Heart Center, Tehran University of Medical Sciences, North Karegar Avenue, Tehran 1411713138, Iran

## Abstract

*Aim*. To demonstrate the prevalence of isolated organisms in urinary/respiratory tract infections and their antibiotic susceptibilities in a tertiary care center. *Methods and Material*. Between January 2008 and January 2010, patients referring to the clinic of cardiology or those admitted to the cardiac wards were enrolled in this cross-sectional descriptive study. Urine and sputum sampling was done for all the patients and the specimens underwent microbiologic examination and, in case of isolation of microorganism, antibiotic disk diffusion test was performed. *Results. Escherichia coli (E. coli)* was the most prevalent isolated organism in-hospital and community-acquired UTIs and was highly resistant to cephalothin in all the samples followed by cotrimoxazole, and ceftriaxone. It revealed high sensitivity to imipenem, amikacin, and nitrofurantoin. *Acinetobacter* constituted the most prevalent organism isolated from respiratory secretions and represented the highest resistance to ceftriaxone and the greatest sensitivity to imipenem. *Conclusions. E. coli* and *Acinetobacter* remain the most common uropathogenic and respiratory organisms, respectively. However, their increasing resistance to wide-spectrum imipenem, meropenem, and vancomycin is a major concern.

## 1. Introduction

With the increasing utility of invasive procedures in inpatient settings, nosocomial infections are encountered with more prevalence among the hospitalized population. Hospital-/noncommunity acquired infections (HAI) not only lengthen the duration of hospital stay with increased demand for additional health care resources but also are potential sources for acquisition of microorganisms by the health care providers, burdens health care system itself, and sets up the transmission into shorter cycle [1, 2]. In essence, the most common pathogens responsible for hospital-acquired infections differ from those of community-acquired infections [2]. On the other hand, antimicrobial susceptibility of each organism is influenced by the origin of infection, periodic alterations, and epidemiological factors of that region [1, 3–5].

Urinary and respiratory tract infections are the leading cause of in-hospital infections consuming huge amounts of health resources. In addition, these infections impose the risk of high morbidity and mortality on admitted patients [6]. Empirical treatment for HAI should be instituted based on the knowledge of susceptibility of responsible pathogens to available antibiotics [5–7].

Although this approach optimizes the effects of antimicrobial agents, inappropriate extended-spectrum antibiotics should strongly be discouraged. The importance is more sensible if increased resistance to many antibiotics is taken into account and action programs have developed to restrict overuse of antibiotics [3, 5, 7–9]. For this reason, specific nationwide surveillance studies should be conducted to provide for microbiological resistance pattern and antibacterial activity of antibiotics. The aim of our study was to provide objective evidence in terms of antibiotic susceptibility and resistance of pathogens isolated from patients afflicted by either community- or noncommunity acquired UTI and RTI in a tertiary care center in Iran.

## 2. Material and Methods

### 2.1. Patients

This is a cross-sectional study conducted from January 2008 to January 2010. The study population included patients attending to the laboratory or admitted to cardiac wards in Tehran Heart Center (THC), Tehran, Iran.

The inclusion criteria comprised clinical evidence of urinary or respiratory tract infection, according to the definition of center for disease control and prevention (CDC), in patients admitted to the hospital before 48 hours of admission and in patients presenting to the outpatient clinic having symptoms for less than 72 hours.

The inclusion criteria comprised community-acquired infections diagnosed in patients attending to the laboratory without prolonged duration (>72 hrs) and previous antibiotics reception and hospital-acquired infections considered in patients developing U/R tract infections more than 48 hours after hospital admission. Admitted patients with documented UTI or RTI on admission or outpatient subjects who had received antibiotic therapy within 48 hours, immune compromised patients, and those with documented complicated infections were excluded from the study. The study design and protocol was approved by the Research and Ethics Committee of THC. Informed consent was documented for each patient at the beginning of the study.

### 2.2. Sampling and Laboratory Assessment

Patients with suggestive symptoms of upper respiratory tract infection or clinical evidence of urinary tract infection underwent sampling for sputum and urine analysis before antibiotic therapy, respectively. Fifty milliliter (mL) of clean-catch midstream urine from patients with a probable diagnosis of UTI was collected in a sterile container for UTI as soon as possible after admission. In those with evidence of an RTI, 20 to 30 mL of early morning sputum was obtained in a sterile plastic container.

Bacteriuria was determined with quantitative technique using 0.01 mL calibrated wire loop to inoculate the blood agar, which provides for growth of most organisms. The same technique was accomplished with the MacConkey culture medium specialized for isolation of gram-negative bacteria. The culture media were incubated at 35 ± 2°C from 18 to 24 hours. Culture was considered positive if at least 10^5^ CFU/mL was isolated for a single organism.

Identification of isolates was obtained by their characteristic appearance on their respective media and gram staining. The isolated colony was confirmed by the pattern of biochemical reactions using the standard method [10] and also by PCR for the type of microorganism [11]. Antibiotic susceptibility was evaluated by Kirby-Bauer disc diffusion method on Mueller-Hinton agar according to the guidelines of Clinical and Laboratory Standards Institute (CLSI) [12] and antibiogram was obtained.

The discs were taken from HiMedia Laboratories (India). The followings are the concentrations of drugs used for disc diffusion testing: ampicillin (10 *μ*g), amikacin (30 *μ*g), coamoxiclav (20/10 *μ*g), cefepime (30 *μ*g), cefoxitin (10 *μ*g), ceftazidime (30 *μ*g), ceftriaxone (30 *μ*g), cephalotin (30 *μ*g), ciprofloxacin (5 *μ*g), clindamycin (2 *μ*g), cotrimoxazole (23.75 *μ*g sulfamethoxazole/1.25 *μ*g trimethoprim), erythromycin (15 *μ*g), gentamicin (10 *μ*g), imipenem (10 *μ*g), meropenem (10 *μ*g), nitrofurantoin (100 *μ*g), penicillin (10 units), tobramycin (10 *μ*g), and vancomycin (30 *μ*g).

### 2.3. Statistical Analysis

Data were assessed using the statistical package for social sciences (IBM SPSS Statistics, version 19, IBM Corporation) and presented as number (percent) and mean ± SD when appropriate.

## 3. Results

Urinary and respiratory samples from admitted patients in 4 intensive care units (ICU), 5 coronary care units (CCU), 12 inpatient wards, and those attended to the laboratory were analyzed. Of 1099 positive cultures, 835 (75.9%) and 264 (24.1%) samples were related to UTI and RTI, respectively.  Table 1 demonstrates annual prevalence of microorganisms with the highest frequency.

### 3.1. Urinary Isolates

Of 835 positive urine cultures, 664 samples (79.5%) were obtained from hospitalized patients compared with 171 samples (20.5%) from outpatient.* E. coli* was the most prevalent isolated organism in both the hospital-acquired and the community-acquired UTIs followed by* Klebsiella* and* Enterococcus* (Table 2). In outpatients,* E. coli* was the leading cause followed by* Klebsiella*,* Staphylococcus epidermidis*, and inactive* E. coli*.

*E. coli* was highly resistant to cephalotin in the majority of samples (56.6%) followed by cotrimoxazole (49.4%) and ceftriaxone (38.5%) as presented in Table 3. Antibiotic susceptibility and resistance pattern of* Klebsiella* and* Enterococcus* species have been also presented in Table 3. It is perceived from the table that the best antibiotics for* Klebsiella* and* Enterococcus* were imipenem and nitrofurantoin, respectively. On the other hand, we found a high susceptibility of* E. coli* to imipenem, amikacin, and nitrofurantoin.  Table 4 summarizes the prevalence of microorganisms isolated from urine samples according to the source of infection and time of sampling.

### 3.2. Respiratory Isolates

Two hundred sixty-four positive cultures were obtained from respiratory secretions. Of these, 135 samples (51.1%) were related to ICU wards compared with 129 samples (48.9%) obtained from hospital wards.* Acinetobacter* constituted the most prevalent organism followed by* Pseudomonas aeruginosa* and* Staphylococcus aureus*. Regarding the admission wards,* Acinetobacter* was the most prevalent organism in the ICU, while* Pseudomonas aeruginosa* was the most prevalent organism in non-ICU wards.* Acinetobacter* demonstrated the greatest resistance to ceftriaxone with the greatest susceptibility and lowest resistance to imipenem.* Pseudomonas aeruginosa* was greatly susceptible to meropenem and highly resistant to ceftazidime.* Staphylococcus aureus* was highly susceptible to vancomycin and highly resistant to erythromycin and cefoxitin, respectively. Susceptibility and resistance to antibiotics of the isolated respiratory organisms are summarized in Table 5.

## 4. Discussion

The importance of regional specification of RTI and UTI etiology and their antibiotic susceptibility has been convincingly proved by several studies [5, 13–18]. However, the knowledge needs to be regularly revised with changing trends in microbiological patterns of the responsible organisms [15]. This accelerates the initiation of adequate antibiotic treatment in nosocomial infection with better results in terms of outcome and improved survival [19].

Our study confirmed that* E. coli* is still the most frequent pathogen in both outpatient and inpatient settings. Although this is in accord with a number of previous reports [13, 16, 17], Muvunyi and colleagues (2011) proposed that the prevalence of urinary infections with* E. coli* is decreasing probably due to other superseding members of the Enterobacteriaceae [15].

The second and the third most prevalent uropathogens in the hospitalized population of our study were* Klebsiella* and* Enterococcus*, respectively. Nevertheless,* Staphylococcus epidermidis* replaced the latter in the outpatient isolates. Similar to other investigations [3],* E. coli* constituted a lower percentage of isolates in inpatients than outpatients. However, the pattern of isolates in an ambulatory setting may be quite variable, with* E. coli* being the most common organism, followed by* Staphylococcus saprophyticus, Proteus mirabilis*, and Enteric gram-negative rods in one study [8]. Another observational study on microbiologic patterns of UTI in general practice also revealed* E. coli* as the most common culprit followed by typical uropathogens and* Enterococcus faecalis* [9]. According to current evidence, irrespective of the chronological pattern of the infection,* E. coli* still accounts for the most common organism isolated from urinary tract infections in either of the inpatient or outpatient populations which should be investigated in terms of antimicrobial susceptibility.

With respect to RTI,* Acinetobacter* constituted the most common pathogen in 2008 followed by* Pseudomonas aeruginosa* and* Staphylococcus aureus*. Nevertheless, the trend has been different in 2007 with* Pseudomonas aeruginosa* being the most prevalent and* Acinetobacter* being the least frequent organisms. In a study by Wang et al. from northern China,* Pseudomonas aeruginosa* was the most common isolated organism in hospitalized patients with lower respiratory tract infection [18]. However, a SENTRY antimicrobial surveillance program showed that* Staphylococcus aureus* comprised 28 percent of isolates followed by* Pseudomonas aeruginosa* in 20.0 percent,* Streptococcus pneumonia* in 9.1 percent, and* Klebsiella saprophyticus* in 7.5 percent among hospitalized patients in northern America and Canada [14]. Xia and colleagues investigated the changing pattern of isolated pathogens in lower respiratory tract infection among a Chinese population and found that* Acinetobacter* was more frequently isolated in 2006 than in 2010 [5] which is also confirmed by our study (15% in 2007 versus 36.8% in 2008). Moreover, it seems that* Acinetobacter* is commonly isolated more from ICU patients than from other wards perhaps due to the compromised immunity of the critically ill patients [5, 18].

The urinary isolated* E. coli* was susceptible to nitrofurantoin in 90 percent and to gentamicin in 71.8 percent of cases. Although susceptibility to imipenem and meropenem remained high (100 percent and 80.4 percent, resp.), cotrimoxazole represented a susceptible proportion of less than 50 percent. Additionally,* Klebsiella* demonstrated 80.7 percent susceptibility to gentamicin, 83.9 percent to ciprofloxacin, 58.5 percent to nitrofurantoin, and more than 60 percent to cephalosporin. However, it raised concern because of resistance to coamoxiclav in 61.8 percent and to ampicillin in 92.6 percent of the isolates. Similarly,* Enterococcus* showed susceptibility to nitrofurantoin (74.1%) and gentamicin (48%) with 55.2 percent resistance to vancomycin, hence, categorized as vancomycin resistant* Enterococcus* (VRE). However, the susceptibility and resistance patterns were relatively comparable so as both features are close to 50 percent. In a study by Cullen and colleagues,* E. coli*, as the most common uropathogen, showed significant resistance to penicillin, trimethoprim, and ciprofloxacin [3]. Their results also showed a decreasing susceptibility to gentamicin during the period of study for 11 years.

Respiratory isolated* S. aureus* showed 100 percent susceptibility to vancomycin. Cotrimoxazole had also over 90 percent activity against this species. In contrast, resistance against cefoxitin and erythromycin exceeded 80 percent implying that more than 80 percent of the organisms were methicillin resistant* S. aureus* (MRSA).* Acinetobacter*, as the most common isolate in RTI, represented the best susceptibility to imipenem (50%). Nevertheless, its resistance to imipenem (47.9%) was relatively too high to be selected as the first line antibiotic. On the other hand, other antibiotics did not reveal high activity against this organism with the best susceptibility varying from 10 to 15 percent (see Table 5). Interestingly,* Pseudomonas aeruginosa* had high susceptibility to available antibiotics such as meropenem (90%), ciprofloxacin (87.5%), imipenem (84.4%), and amikacin (73.4%). In their study, Jones et al. rated antimicrobial resistance of pathogens in pediatric infections and reported that ceftriaxone and cefepime were generally more effective against* E. coli* and* Klebsiella pneumonia* [4]. However, imipenem was the most effective antibiotic with 100 percent susceptibility. These findings confirm that despite current trends in pediatric population, where administration of imipenem is not widely established yet, the community-acquired pathogens are still amenable with high susceptibility to this antimicrobial agent.

The present study was employed as surveillance of current microbiologic pattern and antibiotic susceptibility in UTI and RTI among a large hospital and outpatient population in order to revise the existing knowledge for the establishment of proper and adequate empirical antibiotic therapy. However, a multicenter investigation would have complied with this objective more sufficiently. Furthermore, the history of infection and previous administration of antibiotics are influential factors for striding towards understanding elements of antimicrobial resistance. On the other hand, the role of other nonbacterial microorganisms in community- and in-hospital acquired infections should be taken into account [20]. Periodic domestic antimicrobial surveillances are needed to regularly update the guidelines on proper empiric and organism-specific antibiotic treatment [7].

## 5. Conclusion

Our study showed the changing trends in antibiotic susceptibility of isolates from UTI and RTI with respect to the source of infection during the time. While* E. coli* and* Acinetobacter* remain the most common urinary and respiratory organisms, respectively, their increasing resistance to wide-spectrum imipenem and meropenem is a major concern.

## Figures and Tables

**Table 1:** Annual prevalence of microorganisms with the highest frequency.

Organism	Urine		Organism	Sputum	
	2007	2008		2007	2008
*E. coli *	249 (64%)	272 (60.9%)	*Pseudomonas aeruginosa *	37 (30.8%)	29 (20.1%)
*Klebsiella *	31 (7.9%)	39 (8.7%)	*Staphylococcus aureus *	29 (24.1%)	26 (18%)
*Enterococcus *	26 (6.6%)	33 (7.3%)	*Acinetobacter *	18 (15%)	52 (36.8%)

**Table 2:** Discriminative prevalence of isolated organism.

Type of organism	Urine sample (*n* = 835)	Sputum sample (*n* = 264)
*Acinetobacter *	5 (0.5%)	71 (26.8%)
*Citrobacter *	10 (1.1%)	4 (1.5%)
*E. coli *	521 (62.3%)	14 (5.3%)
*E. coli inactive *	37 (4.4%)	1 (0.4%)
*Enterobacter *	23 (2.7%)	11 (4.1%)
*Enterococcus *	59 (7%)	2 (0.7%)
*Klebsiella *	70 (8.3%)	11 (4.1%)
*Proteus *	6 (0.7%)	0 (0)
*Pseudomonas aeruginosa *	17 (2%)	66 (25%)
*Staphylococcus aureus *	12 (1.4%)	55 (20.8%)
*Staphylococcus epidermidis *	29 (3.4%)	16 (6%)
*Staphylococcus haemolyticus *	11 (1.3%)	5 (1.8%)
*Staphylococcus saprophyticus *	2 (0.2%)	1 (0.4%)
*Streptococcus* group A	0 (0)	2 (0.7%)
*Streptococcus* group D	0 (0)	1 (0.4%)
*Streptococcus saprophyticus *	33 (3.9%)	0 (0)

**Table 3:** Antimicrobial susceptibility pattern of isolated organisms from urine samples.

Organism	Type of antibiotic	Susceptibility level		
		Susceptible	Intermediate	Resistant
*E. coli *	Imipenem	514 (100%)	0 (0%)	0 (0%)
	Amikacin	483 (94.2%)	10 (1.9%)	20 (3.9%)
	Nitrofurantoin	445 (90.1%)	22 (4.5%)	27 (5.5%)
	Gentamicin	370 (71.8%)	23 (4.5%)	122 (23.7%)
	Cefepime	322 (63%)	25 (4.9%)	164 (32.1%)
	Ceftazidime	301 (58.7%)	21 (4.1%)	191 (37.2%)
	Ceftriaxone	289 (57%)	23 (4.5%)	195 (38.5%)
	Ciprofloxacin	286 (55.6%)	35 (6.8%)	193 (37.5%)
	Cotrimoxazole	224 (47.1%)	17 (3.6%)	235 (49.4%)
	Cephalothin	145 (28.4%)	77 (15.1%)	289 (56.6%)

*Klebsiella *	Imipenem	57 (100%)	0 (0%)	0 (0%)
	Meropenem	26 (100%)	0 (0%)	0 (0%)
	Amikacin	55 (96.4%)	0 (0%)	2 (3.5%)
	Ciprofloxacin	47 (83.9%)	0 (0%)	9 (16.1%)
	Gentamicin	46 (80.7%)	1 (1.8%)	10 (17.5%)
	Cefepime	41 (71.9%)	3 (5.3%)	13 (22.8%)
	Ceftazidime	36 (65.5%)	1 (1.8%)	18 (32.7%)
	Ceftriaxone	35 (63.6%)	4 (7.3%)	16 (29.1%)
	Cotrimoxazole	34 (63%)	2 (3.7%)	18 (33.3%)
	Nitrofurantoin	31 (58.5%)	8 (15.1%)	14 (26.4%)
	Cephalothin	30 (54.5%)	4 (7.3%)	21 (38.2%)
	Ampicillin	3 (5.6%)	1 (1.9%)	50 (92.6%)
	Coamoxiclav	15 (27.3%)	6 (10.9%)	34 (61.8%)

*Enterococcus *	Nitrofurantoin	40 (74.1%)	5 (9.3%)	9 (16.7%)
	Gentamicin	22 (47.8%)	3 (6.5%)	21 (45.7%)
	Vancomycin	26 (44.8%)	0 (0%)	32 (55.2%)
	Cotrimoxazole	16 (38.1%)	2 (4.8%)	24 (57.1%)
	Ciprofloxacin	19 (35.2%)	16 (29.4%)	19 (35.2%)
	Erythromycin	16 (27.6%)	8 (41.4%)	34 (58.6%)
	Clindamycin	3 (5.9%)	0 (0%)	48 (94.1%)

**Table 4:** Prevalence of microorganisms isolated from urine samples according to source of infection and time of sampling.

	Admission		Source of infection	
	Outpatient	Inpatient	Nosocomial	Community acquired
*E. coli *	121 (70.7%)	400 (60.2%)	79 (53%)	321 (62%)
*Klebsiella *	21 (12.2%)	49 (8.7%)	22 (14.7%)	38 (7.3%)
*Enterococcus *	0 (0)	54 (8.1%)	16 (10.7%)	36 (6.9%)
*Staphylococcus epidermidis *	7 (4%)	0 (0)	0 (0)	0 (0)

**Table 5:** Antimicrobial susceptibility pattern of isolated organisms from sputum.

Organism	Type of antibiotic	Susceptibility level		
		Susceptible	Intermediate	Resistant
*Staphylococcus aureus *	Vancomycin	55 (100%)	0 (0%)	0 (0%)
	Clindamycin	11 (25.6%)	0 (0%)	32 (74.4%)
	Ciprofloxacin	8 (24.2%)	12 (36.4%)	13 (39.4%)
	Erythromycin	8 (15.4%)	0 (0%)	44 (84.6%)
	Gentamicin	4 (13.3%)	3 (10%)	23 (76.7%)
	Cotrimoxazole	46 (90.2%)	0 (0%)	5 (9.8%)
	Cefoxitin	7 (13.3%)	2 (3.8%)	43 (82.7%)

*Acinetobacter *	Imipenem	36 (50.7%)	1 (1.4%)	34 (47.9%)
	Meropenem	8 (17.8%)	2 (4.4%)	35 (77.8%)
	Gentamicin	11 (15.9%)	1 (1.4%)	57 (82.6%)
	Tobramycin	6 (14.3%)	2 (4.8%)	34 (81%)
	Ciprofloxacin	10 (14.1%)	7 (9.9%)	54 (79.1%)
	Amikacin	7 (10.4%)	2 (3%)	58 (86.6%)
	Cotrimoxazole	5 (9.3%)	0 (0%)	49 (90.7%)
	Ceftazidime	5 (7.4%)	1 (1.5%)	62 (91.2%)
	Ceftriaxone	2 (3.2%)	3 (4.8%)	58 (92.1%)

*Pseudomonas aeruginosa *	Meropenem	19 (90.5%)	1 (4.8%)	1 (4.8%)
	Ciprofloxacin	49 (87.5%)	4 (7.1%)	3 (5.4%)
	Imipenem	54 (84.4%)	4 (6.3%)	6 (9.4%)
	Amikacin	47 (73.4%)	5 (7.8%)	12 (18.8%)
	Gentamicin	41 (67.2%)	1 (1.6%)	19 (31.1%)
	Tobramycin	36 (66.7%)	2 (3.7%)	16 (29.6%)
	Ceftazidime	21 (34.4%)	1 (1.6%)	39 (63.9%)
